# The Relationship between Fear of COVID-19 and Self-Medication and the Rate of Antibiotic Use in Patients Referred to COVID-19

**DOI:** 10.1155/2022/3044371

**Published:** 2022-12-09

**Authors:** Fatemeh Faraji, Rostam Jalali, Nader Salari

**Affiliations:** ^1^Department of Medical Surgical Nursing, Kermanshah University of Medical Sciences, Kermanshah, Iran; ^2^Department of Nursing, Kermanshah University of Medical Sciences, Kermanshah, Iran; ^3^Kermanshah University of Medical Sciences, Kermanshah, Iran

## Abstract

**Introduction:**

The coronavirus pandemic can cause anxiety and stress among people, which can make them practice self-medication. This study aimed to investigate the relationship between fear of corona and self-medication and antibiotics use.

**Methods:**

In a convenience sampling method, 250 people referring to COVID-19 centers including 16-hour comprehensive health services in Kermanshah, Iran, who tested positive and were not hospitalized were extracted from the SIB system. Data collection tools include three questionnaires including corona fear questionnaire, self-medication questionnaire, and self-medication by antibiotic questionnaire and an information form including demographic characteristics. Data were analyzed by SPSS version 25.

**Results:**

The prevalence of self-medication was 59.6%. There was a significant correlation between self-medication and gender (*P* value <0.05) and education level (*P* value <0.05); the most common reason for self-medication was easy access to medicines through pharmacy drug stores. The mean score of fear of corona was higher in women and those who were not in a good financial position due to a lack of suitable economic status to see a doctor.

**Conclusion:**

There was a direct and significant relationship between self-medication and self-medication by antibiotics. 59.6% of the participants used medicines themselves, buying over-the-counter in pharmacies. Also, there was a statistically significant difference between the mean score of corona fear in terms of not having a suitable economic status to see a doctor. This indicates that the lack of proper economic status among people with the coronavirus positive test to see a doctor increases the fear of the disease.

## 1. Introduction

Coronaviruses (CoVs) are a large family of viruses, ranging from the common cold virus to the cause of more serious illnesses such as severe acute respiratory syndrome (SARS), Middle East respiratory syndrome coronavirus (MERS-CoV), and COVID-19. The COVID-19 pandemic is an ongoing global health crisis that currently causes severe and acute respiratory distress syndrome in some patients and may lead to the death of some patients [1]. The recent COVID-19 pandemic is in fact a major social event that has been raised not only in one region but also in the country and even the whole world, and it is necessary to consider its social effects [2].

Fear and anxiety caused by a possible illness are destructive and can lead to mental disorders and stress in people [3]. One of the types of people's fears is the fear of getting sick [4]. Because there is still no definitive cure for coronavirus, many people around the world are afraid of getting it. People who are afraid of getting sick constantly think that anything can cause them to get sick, and as a result, they constantly see a doctor or seek self-medication [5].

Self-medication is a process that involves preparation and use of medications without a doctor's recommendation for diagnosis or treatment [6]. The results of published articles show that the prevalence of “self-medication” in Iran is 4 times more than the global average [7]. Antibiotics have the highest rate of arbitrary use. A study on the arbitrary use of herbal and chemical drugs in Isfahan showed that 86% of women had used drugs arbitrarily for at least one disease in the past 6 months [8]. Self-medication in Iran has been increasing in recent years, and about 83% of Iranians living in Tehran practiced self-medication [9].

Self-medication with antibiotics (SMI) and other medications is a problem that increases each year [10]. With the approach of the cold seasons of the year, the risk of catching the flu increases, an issue that is always emphasized by experts because self-administration of antibiotics can increase microbial resistance. With the outbreak of the corona epidemic, due to the similarity of the symptoms of the virus with the flu, colds, and respiratory infections, some people may think that antibiotics are the cure. A study of antibiotic use in developing countries found that people identified antibiotics as an “extraordinary drug” or “a powerful drug” that could prevent and treat any disease [11].

Misconceptions and lack of awareness about antibiotic use have been reported by various studies in developed and developing countries [12]. The World Health Organization (WHO) is concerned that the misuse of antibiotics during the coronavirus crisis will exacerbate this trend.

Limited research has been conducted on anxiety experiences in patients with severe respiratory symptoms. Anxiety can cause people to be unable to distinguish between right and wrong information, so they may be exposed to false news [13]. Due to the rapid spread of the disease and the increasing number of patients and deaths, the fear of getting the disease in the community is increasing, which may lead to self-medication and irrational use of medicines without consulting a doctor. This study also aimed to investigate the relation between fear of getting infected with coronavirus and self-medication among people living in Kermanshah, Iran.

## 2. Methods

This study was conducted using a cross-sectional research plan. The data collected from the dataset and files of patients between 10 July and 20 December 2021 were extracted. 250 people referred to corona centers in Kermanshah, Iran, whose polymerase chain reaction (PCR) test was positive and were not hospitalized were studied. The data were extracted from the SIB system (the latest electronic health record system in Iran). The inclusion criteria was having positive PCR test for corona during the last two weeks with no need for hospitalization. The participants were selected from the 16-hour comprehensive health services in the SIB system. All information about patients would be recorded in the SIB system. The sample size was calculated based on the study of Nasir et al. [14] using the sample size equation with a confidence level of 0.95% and an estimation error of 5%. Incomplete questionnaires were excluded from the study.

Data collection tools included three questionnaires and a demographic form: fear of corona, self-medications, and self-medication with antibiotics. The demographic form included 8 questions about demographic characteristics (age, sex, marital status, economic status, level of education, employment status, and underlying disease).

The corona fear questionnaire was a 5-item test that measured the degree of corona fear on a 5-point Likert scale (1 representing very low to 5 representing very high). This questionnaire was designed in the spring of 1399 by Veisi et al. to evaluate the psychometric properties of the short-term fear of coronavirus disease and its validity and reliability [15].

The self-medication questionnaire was a 13-item test that includes information on over-the-counter medication, causes of self-medication, medications, forms, and frequency of use. This questionnaire was used by Tabiei et al. In a study entitled “Self-medication with drugs in students of Birjand universities,” the validity of its content was confirmed by 5 faculty members of Birjand University of Medical Sciences [16].

The questionnaire of the self-medication with antibiotics included questions about the use of antibiotics in the last three months, how to prepare antibiotics, the symptom that started the use of antibiotics, keeping antibiotics at home, using antibiotics without a doctor's prescription, willingness to keep antibiotics at home or use them without a doctor's prescription, and the reason for the arbitrary use of antibiotics. This questionnaire was used by Alipour et al. and colleagues in a study entitled “The relationship between personality traits and the arbitrary use of antibiotics in students” [17].

After getting the permission of the Vice Chancellor for Research at Kermanshah University of Medical Sciences, data collections were done from May to July 2021. The purpose of the study was explained to the subjects and they were assured about the confidentiality of their personal information and responses to questions. Written informed consent was obtained from all subjects after receiving an explanation of the study. This study has been registered with the Ethics Committee of Kermanshah University of Medical Sciences with the ethics code IR.KUMS.REC.1400.030.

Data analysis was performed using SPSS software version 25. Descriptive statistical methods were used to summarize and describe the variables. The Kolmogorov–Smirnov test was used to evaluate the normality of the distribution of scores of the studied indices. The obtained data were analyzed at a level of significance less than 0.05. The Pearson correlation test (Spearman correlation) was used to examine the correlation between fear score, self-medication, and antibiotic use.

## 3. Results

A total of 250 people participated in this study. The mean age of patients was 31.7 years with a standard deviation of 11.01 years; 47.2% were female, 54% were single, 68% had a university degree, and 91.2% had no underlying disease (Table 1).

The results showed that 63.2% were familiar with the concept of drug resistance. 59.6% of the participants practiced self-medication. 43.6% used the medicine themselves due to the lack of need to see a doctor (Table 2).

40.4% of people considered the insignificance of the disease and 40.4% of the inability to diagnose the symptoms of the disease as the cause of using antibiotics (Table 3).

According to the findings of Table 4, the findings of the Mann–Whitney test showed that there was a statistically significant difference between the mean score of corona fear in terms of not having a suitable economic status to see a doctor (*P* < 0.05).

According to Table 5, the findings of the Spearman correlation test showed that there was a statistically direct and significant relationship between self-medication and self-medications with antibiotics (*P* < 0.05).

The result showed that there are a significant relationship between self-medication and gender (Table 6).

The correlation between the three variables of corona fear, self-medication, and self-medication with antibiotics with demographic variables (age, sex, marital status, economic status, education, employment status, and underlying disease) is given in Table 6.

## 4. Discussion

The results showed that 59.6% of the participants who had a corona positive test practiced self-medication. A previous study that examined the frequency of self-medication in medical students studying at Rafsanjan University of Medical Sciences concluded that approximately 70% of students did self-medication [18]. Another study conducted by Korani et al. 2016 [19] in Kermanshah on assessment of self-medication and associated factors among elderly living in Kermanshah city in 2014 showed that about 90% of the subjects had used self-medicines.

Other studies have reported a prevalence of more than 60% of self-medication: about 71.7% in Kasulkar and Gupta' studies [20], 92% in Badiger et al.' studies [21], and 74% in Zafar' studies [22]. The results of the present study on the frequency of self-medication with antibiotics are consistent with other studies conducted in Iran and the Middle East.

The results of this study and its comparison with other results showed that self-medication is a major problem among people. The issue of medicine use is something that is related to all cultural, social, and economic aspects of people. Self-medication is not a personal issue, and most sections of society are involved in it. People who have tried self-medication and faced side effects and been referred to medical centers do not acknowledge this issue, so health centers cannot provide accurate statistics of people who have tried to practice self-medication, and therefore, the relevant agencies cannot make the necessary plans in this regard. Culture and education have an important role in self-medication. We expect that self-medication rate will decrease by the awareness of the people and the elimination of misconceptions.

The results showed that there was a direct and significant statistical relationship between self-medication and SMI (*P* < 0.05), which shows the tendency of individuals to use antibiotics. People who self-medicated said that the reason for this was the existence of free medicine market in the country (about 43.2%) and the conclusion from previous consumption. A similar study conducted by Zeinali et al. in Tehran showed that about half of participants in the study did self-medication [23]. Furthermore, 52.8% continue to refill their last prescription rather to visit a doctor. Another study conducted among college students in Tehran showed that 53% of students practiced self-medication with antibiotics [24]. This is an alarm for the healthcare system because it shows access to any medicine in the country can be easily done in pharmacies.

The results showed that self-medication with antibiotics was associated with gender and education. Antibiotic self-medication was higher in women than men and was more in people with at least a diploma and university education. Similar results were obtained in previous studies, for example, a study conducted by Jafari et al. on the self-medication in Kermanshah. Self-medication was significantly higher in women and people with university education [25]. In other studies, self-medication was also higher among educated people [26, 27]. Educated people seem to believe that they can get all the information they need from their medicine brochures or previous prescriptions; therefore, they diagnose their disease and start self-medication according to their previous treatment.

The main source that people declare for supplying the medicines for self-medication was pharmacy drug stores [28], which are consistent with the results of this study. This shows that selling over-the-counter drugs has a significant impact on the high prevalence of self-medication.

Also, a study conducted by Tabiei et al. [16] showed that antibiotics (53.1%) are the most commonly used drugs arbitrarily after analgesics. This amount needs more reflection and more careful study because the irrational use of these drugs will increase bacterial resistance, exposure to side effects, and increase treatment costs.

In other studies, the reasons that were declared for self-medication were factors such as keeping drugs at home, believing that the self-medication is safe, not having enough time to see a doctor to solve the problem, delivery of the medicines by pharmacies without a doctor's prescription [29, 30], the cost of doctors' visits, the lack of access to doctors due to financial poverty and high cost of doctors' visits, not covered by health insurance [31, 32], the lack of accurate information about the effects of the medicines, also lack of trust in doctors' medicine [33], lack of need to refer to the physician, and the fear and embarrassment of medical examinations, the crowds of doctors' offices or medical centers have been mentioned as factors related to self-medication [34]. It seems that the cheap price of the medicines also affects its excessive use [35].

The results showed that there was a statistically significant difference between the mean score of corona fear in terms of inexperience and insufficient knowledge of physicians in treatment according to the patient (*P* < 0.05). There was no significant relationship between other components of arbitrary drug use (self-medication) and fear of corona disease.

Findings showed that the level of fear of corona was significantly related to gender; the average score of fear of corona in women was much higher than in men. In a study done by Wang et al. [36], female gender was an important factor in the negative psychological impact of the prevalence of COVID-19. The study included 1,210 respondents from 194 cities in China. According to the authors, women suffered more from the psychological impact of corona disease and also face higher levels of stress, anxiety, and depression. In Italy, results have also been reported to show greater psychological vulnerability associated with COVID-19 in women. The results of this study showed that women are significantly more likely to have higher levels of stress, anxiety, insomnia, and depression [37]. The fact that women are more afraid than men during COVID-19 may be related to several factors. Some factors may be immediate and others may have long-term consequences. Fear may be caused by predicting the negative impact of the disease on the health of the individual and the health of family and close friends. On the other hand, the closure of schools and kindergartens has greatly increased the need for child care, which has a significant impact on working mothers. This result addresses the need to design interventions that reduce the negative impact of the current prevalence on women's mental health. This may also be explained by the fact that women have shown exaggerated emotional reactions and negative emotions in response to stressful situations and are usually more burdened than men during the epidemic, including housework, caring role, or they have domestic violence. In addition, women are more likely than men to suffer from stressful life events.

The mean score of corona fear was higher among employed people and students. These results were generally in line with the results of other studies. In a study conducted in Bangladesh, the results showed that more than two-thirds of students experienced mild-to-severe depression (82.4%) and anxiety (87.7%) [38]. Therefore, more psychological evaluation and even, in some cases, intervention are needed.

Another study conducted in China examined the psychological effects of the epidemic on students. The results showed the corona's economic effects and its effect on daily life, such as delays in academic activities, increased anxiety, and fear symptoms among students [39]. Other studies have concluded that the prevalence of corona disease has a significant effect on work stress and that employed people are more affected by the consequences of the epidemic. In other words, physical, psychological, financial, and social concerns about infectious diseases cause work stress, which negatively affects the overall performance of employees [40].

## 5. Conclusions

The results of this study showed that the arbitrary use of the drug for self-medication rate is significantly high and the sale of over-the-counter antibiotics by pharmacies and the lack of sufficient information provided to patients about the correct use of the drug is a medical problem. The mean score of corona fear and the rate of self-medication with antibiotics were higher in women than men, and the rate of self-medication was higher in people with higher education. Also, there was a statistically significant difference between the mean score of corona fear in terms of not having a suitable economic status to see a doctor. This indicates that the lack of proper economic status of people with a positive corona test to see a doctor increases the fear of corona disease.

## Figures and Tables

**Table 1:** Distribution of relative and absolute frequencies of demographic variables in research units.

Variables		Percent	Frequency
Gender	Female	47.2	118
	Male	52.8	132

Marital status	Single	54.4	136
	Married	45.6	114

Housing situation	Rented	21.2	53
	Private	30.8	77
	With family	45.2	113
	Others	2.8	7

Job	Employed	65.3	134
	Household	11.6	29
	Student	16.4	41
	No job	18.4	46

Income	Weak	24.4	61
	Medium	62	155
	Good	13.6	34

Education	Lower than diploma	4	10
	Diploma	28	70
	Higher than diploma	68	170

Underlying disease	Yes	8.8	22
	No	91.2	228

**Table 2:** Distribution of relative and absolute frequency of arbitrary drug consumption in research units.

Reasons for self-medication	No	Yes
	Percent (frequency)	Percent (frequency)
Familiarity with the concept of drug resistance	36.8 (92)	63.2 (158)
Self-medication	40.4 (101)	59.6 (149)
Due to the lack of need to see a doctor	56.4 (141)	43.6 (109)
Not having enough time to see a doctor	63.2 (158)	36.8 (92)
To avoid paying for a doctor's visit	74.4 (186)	25.6 (64)
Due to not having a health insurance	80.4 (201)	19.6 (49)
Due to the safety of the drugs used	65.6 (164)	34.4 (86)
Due to getting the desired result from the previous arbitrary consumption	60.8 (152)	39.2 (98)
Due to the preference of foreign drugs over Iranian	75.2 (188)	24.8 (62)
To store medicine at home for later use	66 (165)	34 (85)
Due to the lack of medication prescribed by some doctors	80.4 (201)	19.6 (49)
Due to not believing in the doctor's treatment	86 (215)	14 (35)
Due to being in a hurry	65.6 (164)	34.4 (86)
Due to lack of feeling the need for a doctor's opinion	70 (175)	30 (75)
Due to not having a suitable financial situation to see a doctor	78.8 (197)	21.2 (53)
Due to the supply of medicine by the pharmacy	69.6 (174)	30.4 (76)
Because of the advice of others	66.4 (166)	33.6 (84)

**Table 3:** Distribution of relative and absolute frequency of antibiotic use in the participants.

Reason for antibiotic use	No	Yes
	Percent (frequency)	Percent (frequency)
Getting results from a previous medication	60.8 (152)	39.2 (98)
The insignificance of the disease	59.6 (149)	40.4 (101)
Inability to diagnose symptoms	59.6 (149)	40.4 (101)
No risk or side effects when taking the drug	68.8 (172)	31.2 (78)
Ease of over-the-counter medication to your doctor	69.2 (173)	30.8 (77)
Existence of a free pharmaceutical market in the country	56.8 (142)	43.2 (108)
Lack of time to see a doctor	64.8 (162)	35.2 (88)
Experience of not delivering the items written in the prescription by the pharmacy	72 (180)	28 (70)
Insufficient experience and knowledge of physicians in treatment according to the patient	72.8 (182)	27.2 (68)
Unable to pay for a doctor's visit	74.8 (187)	25.2 (63)
Lack of insurance	80.8 (202)	19.2 (48)
Restrictions on seeing a doctor (doctor not available)	77.6 (194)	22.4 (56)
Failure to see a doctor due to illness due to worsening of the disease	90.8 (227)	9.2 (23)

**Table 4:** Distribution of relative and absolute frequency of self-medication in the participants.

Questions	Test statistic	No	Yes	*P* value
		Mean ± SD	Mean ± SD	
Familiarity with the concept of drug resistance	−1.3	4.3 ± 12.2	4.2 ± 11.4	0.177
Self-medication	−0.615	4.4 ± 11.9	4.2 ± 11.5	0.539
Due to the lack of need to see a doctor	−1.2	4.4 ± 11.4	4.1 ± 12.08	0.203
Due to not having enough time to see a doctor	−0.494	4.3 ± 11.6	4.2 ± 11.8	0.622
To avoid paying for a doctor's visit	−0.971	4.2 ± 11.5	4.5 ± 12.1	0.331
Due to not having a health insurance	−1.5	4.2 ± 11.5	4.5 ± 12.5	0.119
Due to the safety of the drugs used	−1.6	4.1 ± 11.4	4.5 ± 12.3	0.107
Due to getting the desired result from the previous arbitrary consumption	−1.6	4.1 ± 11.3	4.4 ± 12.3	0.092
Due to the preference of foreign drugs over Iranian	−0.197	4.3 ± 11.7	4.3 ± 11.7	0.844
To store medicine at home for later use	−1.1	4.3 ± 11.5	4.3 ± 12.1	0.267
Due to the lack of medication prescribed by some doctors	−0.342	4.2 ± 11.7	4.4 ± 11.5	0.732
Due to not believing in the doctor's treatment	−0.625	4.3 ± 11.8	4.2 ± 11.2	0.532
Due to being in a hurry	−1.2	4.3 ± 11.5	4.2 ± 11.2	0.225
Due to lack of feeling the need for a doctor's opinion	−0.186	4.3 ± 11.7	4.3 ± 11.6	0.853
Due to not having a suitable financial situation to see a doctor	−2.4	4.1 ± 11.4	4.6 ± 13	0.016^*∗*^
Due to the supply of medicine by the pharmacy	−0.430	4.1 ± 11.5	4.6 ± 12.1	0.789
Because of the advice of others	−0.750	4.6 ± 12.08	4.1 ± 11.5	0.453

**Table 5:** Correlations between self-medication, fear of corona, and antibiotic use.

Variables	Fear of corona		Self-medication		Self-medication with antibiotics	
	*P* value	Spearman correlation coefficient	*P* value	Spearman correlation coefficient	*P* value	Spearman correlation coefficient
Self-medication	0.255	−0.072		1	0.001^*∗*^	0.624
Fear of corona		1	0.255	−0.072	0.319	−0.063
Self-medication with antibiotics	0.319	−0.063	0.001^*∗*^	0.624		1

**Table 6:** Measuring the relationship between the three variables of corona fear, self-medication, and self-medication with antibiotics with demographic variables (age, sex, marital status, economic status, education, employment status, and underlying disease).

Variables		Self-mediation with antibiotics	Fear of corona	Self-medication
		Mean ± SD	Mean ± SD	Mean ± SD
Gender	Female	22.7 ± 2.8	12.4 ± 4.2	28.9 ± 4.6
	Male	21.7 ± 3.4	11.1 ± 4.3	27.8 ± 5.1
*P* value		**0.017** ^*∗*^	**0.020** ^*∗*^	0.101

Marital status	Single	22.1 ± 3.07	11.8 ± 4.2	28.8 ± 4.8
	Married	22.3 ± 3.2	11.6 ± 4.4	27.8 ± 5.1
*P* value		0.427	0.597	0.167

Status of housing	Rent	22.2 ± 3.04	11.8 ± 5.1	28.1 ± 4.8
	Private	22.09 ± 3.4	11.6 ± 3.9	28.06 ± 5
	With family	22.3 ± 2.9	11.7 ± 4.2	28.7 ± 4.8
	Others	20.4 ± 3.2	12 ± 3.7	26.4 ± 6.5
*P* value		0.959	0.964	0.572

Job	Employed	22.3 ± 3.04	10.9 ± 4.1	28.6 ± 4.7
	Household	22.2 ± 2.7	12.8 ± 4.4	27.2 ± 5.09
	Student	22.1 ± 3.4	13.1 ± 4.2	29.04 ± 5.1
	No job	21.8 ± 3.4	11.9 ± 4.4	27.6 ± 5.3
*P* value		0.880	**0.009** ^*∗*^	0.307

Income	Weak	21.5 ± 3.4	13.01 ± 5.1	26.8 ± 5.3
	Medium	22.4 ± 3.07	11.4 ± 3.9	28.8 ± 4.5
	Good	22.5 ± 2.8	10.8 ± 3.6	28.7 ± 5.4
*P* value		0.156	**0.072**	0.030^*∗*^

Education	Lower than diploma	19.2 ± 2.7	13.4 ± 4.4	24.9 ± 4.7
	Diploma	22.4 ± 3.09	12.3 ± 4.6	29 ± 4.8
	University education	22.3 ± 3.1	11.4 ± 4.1	28.3 ± 4.9
*P* value		**0.010** ^*∗*^	0.238	0.039^*∗*^

Underlying disease	Yes	19.6 ± 2.4	12.5 ± 3.9	24.9 ± 4.7
	No	22.5 ± 3.09	11.6 ± 4.3	28.6 ± 4.8
*P* value		**0.001** ^*∗*^	0.402	0.001^*∗*^

## Data Availability

The data used to support the findings of this study are available from the corresponding author upon request (ks_jalali@yahoo.com).
